# Cigarette smoking associates with body weight and muscle mass of patients with rheumatoid arthritis: a cross-sectional, observational study

**DOI:** 10.1186/ar2429

**Published:** 2008-05-20

**Authors:** Antonios Stavropoulos-Kalinoglou, Giorgos S Metsios, Vasileios F Panoulas, Karen MJ Douglas, Alan M Nevill, Athanasios Z Jamurtas, Marina Kita, Yiannis Koutedakis, George D Kitas

**Affiliations:** 1 School of Sport, Performing Arts & Leisure, Wolverhampton University, Gorway Road, Walsall, WS1 3BD, West Midlands, UK; 2 Research Institute in Healthcare Science, University of Wolverhampton, Wulfruna Street, Wolverhampton, WV1 1LY, West Midlands, UK; 3 Department of Rheumatology, Dudley Group of Hospitals NHS Trust, Russell's Hall Hospital, Pensnett Road, Dudley, DY1 2HQ, West Midlands, UK; 4 Department of Sport and Exercise Science, University of Thessaly, Trikala-Karyes Road, Trikala, 42100, Greece; 5 Institute of Human Performance & Rehabilitation, Trikala-Karyes Road, Trikala, 42100, Greece; 6 ARC Epidemiology Unit, University of Manchester, Oxford Road, Manchester, M13 9PT, UK

## Abstract

**Introduction:**

Rheumatoid arthritis (RA) is associated with altered metabolism leading to muscle wasting. In the general population, cigarette smoking is known to affect body composition by reducing fat and inhibiting muscle synthesis. Even though smoking has been implicated in the pathophysiology and progression of RA, its possible effects on body composition of such patients have not been studied. This cross-sectional study aimed to identify potential associations of smoking with body weight and composition of RA patients.

**Methods:**

A total of 392 patients (290 females) with RA were assessed for body mass index (BMI), body fat (BF), fat-free mass (FFM), and waist circumference. Erythrocyte sedimentation rate, C-reactive protein, Disease Activity Score-28, and Health Assessment Questionnaire score were used to assess disease activity and severity. Smoking habit (current smoker, ex-smoker, or never-smoker) and intensity (pack-years) were also noted.

**Results:**

Current smokers had a significantly lower BMI compared with ex-smokers (mean difference: male -2.6, 95% confidence interval [CI]: -3.5 to -1.7; female: -2.6, 95% CI: -4.8 to -0.5) and never-smokers (mean difference: male -1.8, 95% CI: -3 to -0.6; female: -1.4, 95% CI: -2.4 to -0.4). Similarly, the BF of current smokers was lower compared with that of ex-smokers (mean difference: male: -4.3, 95% CI: -7.5 to -1.2; female: -3.4, 95% CI: -6.4 to -0.4) and never-smokers (mean difference: male: -3.3, 95% CI: -6.3 to -0.4; female: -2.1, 95% CI: -4 to -0.2). FFM did not differ between groups. Finally, current smokers had a significantly smaller waist circumference compared with ex-smokers only (mean difference: male: -6.2, 95% CI: -10.4 to -1.9; female: -7.8, 95% CI: -13.5 to -2.1). Following adjustments for age, disease duration, and HAQ score, smoking remained a significant predictor for BMI (*P *< 0.001), BF (*P *< 0.05), and waist circumference (*P *< 0.05). Pack-years were inversely correlated with BF (*r *= -0.46; *P *< 0.001), and heavy smokers exhibited a significantly lower FFM (*P *< 0.05) compared with all other participants.

**Conclusion:**

Within the limitations of a cross-sectional study, it appears that cigarette smoking associates with reduced BMI and BF in patients with RA and heavy smoking associates with lower muscle mass. Smoking cessation appears to associate with increased BMI, BF, and waist circumference in these patients. These results should be confirmed in prospective studies. Given the numerous adverse effects of smoking on general health and RA, patients should be actively advised against it. However, smoking cessation regimes in RA may need to include more general lifestyle counselling, particularly about weight control.

## Introduction

Rheumatoid arthritis (RA), the commonest inflammatory arthritis, is associated with altered metabolism [1]. Compared with healthy controls, RA patients exhibit elevated resting energy expenditure (REE) and enhanced muscle catabolism [2]. Such changes may lead to rheumatoid cachexia (that is, involuntary loss of fat-free mass [FFM] with a proportional increase of body fat [BF]) in the presence of stable body weight [3,4]. Body composition changes, particularly BF increase, may remain largely undetected by traditional assessments such as the body mass index (BMI) [5]. Increased BF, together with reduced levels of physical activity due to joint inflammation and damage [3,6], is associated with several comorbidities, including cardiovascular disease [7,8] as well as increased mortality [3].

Cigarette smoking is an important risk factor for several diseases [9]. It is also known to decrease body weight in healthy individuals by reducing appetite and increasing REE [10]. In contrast, smoking cessation may associate with significant weight increase, which constitutes a major deterrent to smoking control [11].

We have recently demonstrated that smoking further increases REE in RA [12] and this could potentially augment rheumatoid cachexia in these patients. Given the RA-related alterations in body composition and the comorbidity associated with them, the examination of potential contributors to muscle wasting, such as smoking, is important. The aim of this cross-sectional study was to detect potential associations between smoking and body weight, body composition, and rheumatoid cachexia in RA patients.

## Materials and methods

### Participants

Consecutive patients attending routine rheumatology clinics at the Dudley Group of Hospitals NHS Trust, UK, were invited to participate. All applicable institutional and governmental regulations concerning the ethical use of human volunteers were followed during this research. The study had local research ethics committee and research and development directorate approvals, and all volunteers provided informed consent. A total of 400 volunteers (108 males and 292 females) with RA (1987 revised American College of Rheumatology criteria [13]) were assessed. Of them, 8 (6 males) were excluded from the analyses due to missing data for body composition. Data from the remaining 392 (median age: 63.1 [55.5 to 69.6] years; median disease duration: 10 [4 to 18] years) were analysed.

### Assessments

All volunteers were subjected to the same data collection procedures overseen by the same trained investigators. Standing height was measured to the nearest 0.5 cm on a Seca 214 Road Rod portable stadiometer (Seca gmbh & co. kg., Hamburg, Germany). Body weight and composition (that is, BF and FFM) were assessed using a Tanita BC- 418 MA Segmental Body Composition Analyzer (Tanita Corporation, Tokyo, Japan). After initial manual entry of their demographic details, participants stood barefooted on the analyzer and held the handgrips provided until the apparatus printed the results. BMI was calculated on the basis of measured height and weight in kilograms per square metre. Waist circumference was also measured. Contemporary disease activity was assessed by the erythrocyte sedimentation rate (ESR), C-reactive protein (CRP), and the Disease Activity Score-28 (DAS28) [14]. The Anglicised version of the 40-item Stanford Health Assessment Questionnaire (HAQ) [15] was used to measure physical dysfunction as a proxy of disease severity. Patients' self-reported smoking status and intensity (that is, pack-years) were noted.

### Data management and analyses

Data were analysed using the Statistical Package for Social Sciences version 15.0 (SPSS Inc., Chicago, IL, USA). A preliminary evaluation of the variables using a Kolmogorov-Smirnov test of normality revealed that none of them required transformation to reach normality. Mean ± standard deviation was calculated for all variables. Differences in BMI, BF, and FFM between smoking groups are presented as mean differences with 95% confidence intervals (CIs).

According to their smoking status, patients were grouped into never-smokers, current smokers, and ex-smokers. Analysis of variance (ANOVA) assessed differences in demographic characteristics, BMI, and body composition between groups for each gender. Analysis of covariance (ANCOVA) was employed to determine whether the differences observed were attributed to smoking status or other confounding factors (for example, gender, age, and disease characteristics).

In the current smoker and ex-smoker groups, further associations between pack-years with BMI and body composition were examined. Thereafter, patients in these groups were divided into quartiles according to pack-years. ANOVA was employed to assess differences in the measured variables between these subgroups. ANCOVA was used to correct for any confounding factors.

Thereafter, patients were grouped according to (a) RA-specific BMI [5] and (b) gender-specific BF [16] thresholds into underweight, normal weight, overweight, and obese. Subsequently, they were grouped based on gender-specific cut-off points for waist circumference [17] into low or high risk and for FFM into low or normal FFM groups [18]. Chi-square analyses were employed to assess differences between smoking groups in the prevalence of overweight, obesity, high risk, and low FFM. For all tests, the level of significance was set at a *P *value of less than 0.05.

## Results

Table 1 illustrates means ± standard deviations and the ANOVA results for all studied parameters. Current smokers had a significantly lower BMI than ex-smokers (mean difference: male -2.6, 95% CI: -3.5 to -1.7; female: -2.6, 95% CI: -4.8 to -0.5) and never-smokers (mean difference: male -1.8, 95% CI: -3 to -0.6; female: -1.4, 95% CI: -2.4 to -0.4). Current smokers also had a significantly lower BF compared with ex-smokers (mean difference: male: -4.3, 95% CI: -7.5 to -1.2; female: -3.4, 95% CI: -6.4 to -0.4) and never-smokers (mean difference: male: -3.3, 95% CI: -6.3 to -0.4; female: -2.1, 95% CI: -4 to -0.2). FFM did not differ between these groups (mean difference: current smokers versus ex-smokers, male: -4.6, 95% CI: -10.7 to 1.6; female: -1.2; 95% CI: -3.8 to 1.4; current smokers versus never-smokers, male: -2.7, 95% CI: -9.2 to 3.9; female: 0.1, 95% CI: -2.4 to 2.4). Current smokers had a significantly smaller waist circumference than ex-smokers (mean difference: male: -6.2, 95% CI: -10.4 to -1.9; female: -7.8, 95% CI: -13.5 to -2.1) but not never-smokers (mean difference: male: -2.9, 95% CI: -10.6 to 4.9; female: -3.9, 95% CI: -9.2 to 1.5). Also, ex-smokers had a larger waist circumference than never-smokers but the difference was significant for males only (mean difference: male: 3.3, 95% CI: 0.4 to 6.3; female: 3.9, 95% CI: -0.4 to 8.1).

**Table 1:** Measured variables of participants classified as current smokers (CS), ex-smokers (XS), and never-smokers (NS)

Gender	Male (n = 102)			Female (n = 290)		
Smoking status	CS	XS	NS	CS	XS	NS

Number	20	50	32	49	97	144
Age, years	58.8 ± 8.1^a^	65.2 ± 9.9^b^	58.8 ± 15	57.4 ± 13.3^a^	64.1 ± 11.2^b^	60.7 ± 11.8
Height, cm	171.3 ± 7.1	174.3 ± 6.9	172.7 ± 7.7	160.9 ± 6.9	160.8 ± 6.8	159.5 ± 6.8
Weight, kg	76 ± 12.9^b, c^	85.8 ± 13.6	84.1 ± 14.8	67.5 ± 14.2^a^	74.8 ± 15.2	69.9 ± 13.6
Body mass index, kg/m^2^	25.8 ± 3.3^b, c^	28.4 ± 3.8	27.6 ± 4.6	26.1 ± 5.5^a, b^	28.6 ± 5.4	27.5 ± 5
Body fat, percentage	24.5 ± 6.4^c, d^	28.8 ± 6.8	27.8 ± 5.6	35.9 ± 7^a, b^	39.2 ± 6.5	38.1 ± 6.7
Fat-free mass, kg	57.2 ± 9.4	61.7 ± 7.7	59.8 ± 10.3	42.5 ± 4.8	43.7 ± 6.1	42.5 ± 6.1
Waist circumference, cm	100 ± 7.9^c^	106.2 ± 10.8^b^	102.9 ± 9.3	90.8 ± 12.8^a^	98.6 ± 13	94.7 ± 12.7
ESR, mm/hour	26.5 ± 20.5	22.8 ± 21.3	20.7 ± 19.7	30.5 ± 26	34.3 ± 32.7^b^	25.5 ± 19.8
C-reactive protein, mg/L	13.3 ± 9.4	16.1 ± 20.4	16 ± 24.3	21.9 ± 23.2^b^	21.4 ± 32.7^b^	11.9 ± 12.5
DAS28	4 ± 0.9	4.1 ± 1.5	3.9 ± 1.6	4.5 ± 1.5	4.3 ± 1.5	4.1 ± 1.2
HAQ score	0.9 ± 0.8	1.4 ± 1	1.1 ± 0.9	1.5 ± 0.9	1.5 ± 0.9	1.5 ± 0.9
Disease duration, years	8.6 ± 7.8	11.9 ± 10.6	14.6 ± 12.7	11.4 ± 9.8	13.5 ± 10.8	13.5 ± 11.1

Values are presented as mean ± standard deviation. ^a^Significant difference compared with XS (*P *< 0.05). ^b^Significant difference compared with NS (*P *< 0.05). ^c^Significant difference compared with XS (*P *< 0.001). ^d^Significant difference compared with NS (*P *< 0.001). DAS28, Disease Activity Score-28; ESR, erythrocyte sedimentation rate; HAQ, Health Assessment Questionnaire.

In ANCOVA with gender and smoking as factors and age, DAS28, HAQ score, and disease duration as covariates, smoking was a significant and independent predictor for BMI (F_2,387 _= 8; *P *< 0.001), BF (F_2,387 _= 4.4; *P *< 0.05), and waist circumference (F_2,387 _= 7.9; *P *< 0.001). Smoking also emerged as a significant predictor of FFM (F_2,387 _= 5.1; *P *< 0.05), but inclusion of BMI as a covariate eliminated the effect of smoking on FFM (*P *> 0.05).

**Figure 1:**
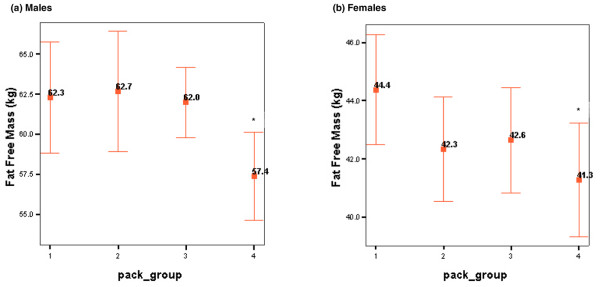
Fat-free mass for males **(a) **and females **(b) **according to pack-year grouping. Data are presented as means with 95% confidence intervals. Pack-year groups: 1, 1 to 9 pack-years; 2, 10 to 19 pack-years; 3, 20 to 34 pack-years; 4, greater than 35 pack-years. Asterisk indicates significant difference compared with group 1 (*P *< 0.05).

There was a significant negative correlation between pack-years and BF (*r *= -0.46; *P *< 0.001) in the current smoker and the ex-smoker groups. This remained significant after adjustment for gender, age, DAS28, HAQ score, and disease duration (F_1,389 _= 4.8; *P *< 0.05). Following pack-year grouping into quartiles (pack-group), ANOVA did not reveal any differences for BMI or body composition among the current and ex-smoker pack-groups. However, an ANCOVA model with gender and pack-group as factors and age and weight as covariates (following stepwise elimination of ESR, CRP, DAS28, HAQ score, and disease duration) revealed a significant effect of pack-group on FFM (F_3,217 _= 2.7; *P *< 0.05), with heavy smokers exhibiting the lowest values. Mean (95% CI) values of this variable in the pack-year subgroups appear in Figure 1.

**Figure 2:**
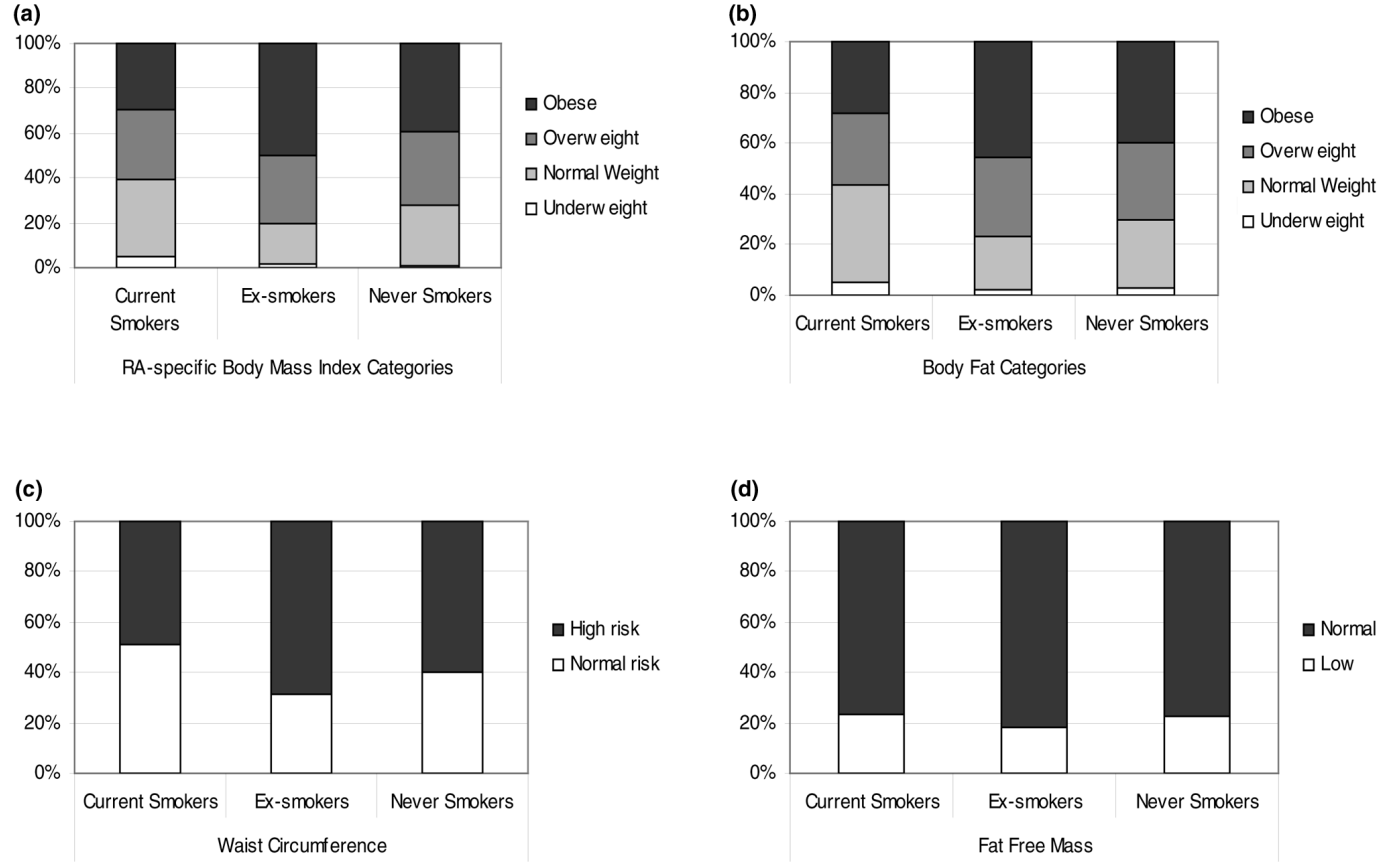
Prevalence of overweight and obesity, increased waist circumference, and low fat-free mass in smoking groups. **(a) **Prevalence of overweight and obesity based on rheumatoid arthritis (RA)-specific body mass index for current, ex-, and never-smokers. **(b) **Prevalence of overweight and obesity based on body fat for current, ex-, and never-smokers. **(c) **Prevalence of high risk based on waist circumference for current, ex-, and never-smokers. **(d) **Prevalence of low fat-free mass for current, ex-, and never-smokers. Chi-square analyses identified significant defences among smoking groups for prevalence of **(a) **overweight and obesity based on body mass index (*P *< 0.05), **(b) **overweight and obesity based on body fat (*P *< 0.05), and **(c) **increased waist circumference (*P *< 0.05). Prevalence of low fat-free mass did not differ between groups (*P *> 0.05).

Following BMI and BF grouping, chi-square analyses showed significant differences (*P *< 0.05) in the prevalence of overweight and obesity among smoking groups, with obesity being more prevalent in ex-smokers (50%) followed by never-smokers (39%) and current smokers (30%). Similarly, ex-smokers had a significantly (*P *< 0.05) higher prevalence of increased waist circumference (69%) compared with never-smokers (60%) and current smokers (49%). However, FFM did not differ between groups (*P *> 0.05) (Figure 2).

## Discussion

To our knowledge, this is the first study to identify significant associations between smoking, body weight, and body composition of RA patients: current smokers had a significantly lower BMI and BF compared with never-smokers. Both BMI and BF were significantly increased in ex-smokers, whereas very heavy smoking appeared to associate with reduced FFM. The study has several potential limitations. These are all cross-sectional associations, and although they can serve for hypothesis generation, they do not provide definitive evidence for causality or directionality: longitudinal studies are required for this. In addition, body composition was assessed by bioelectrical impedance. This method has been validated [19-23] and is thought to be suitable for body composition studies in diverse populations [22-25], correlates well with the 'gold standards' of dual-energy x-ray absorptiometry and hydrostatic weighing [23], and is widely used in RA research [5,12,24,26,27], but it has not actually been specifically validated in the RA population. Finally, although self-report of smoking, especially smoking history, is generally reliable, both under- and over-reporting can occur [28]. This is unlikely to have influenced the primary findings of this study (that is, the differences between current, ex-, and non-smokers), while any misreporting in pack-years may have been smoothed by the large number of participants. It is difficult to assess any other selection bias: the prevalence of current, ex-, and non-smokers among the participants of this study was similar to that reported for local general population subjects of similar age [29], although it was different from an RA cohort established more than 10 years ago [30].

Our observations for BMI are consistent with those in the general population. Both male and female smokers tend to have decreased BMI compared with their non-smoking counterparts [10,31]. In contrast, significant BMI increases have been noted after smoking cessation [11]. Smokers have increased levels of leptin [32], which regulates food intake and fat deposition [33], and reduced hypothalamic neuropeptide Y [34], which regulates appetite [35]. Smoking-induced increases in the levels of epinephrine, norepinephrine, and thyroid hormones lead to increased energy expenditure at rest [36,37] and during light physical activity [38-40]. However, these effects are short-lived: after smoking cessation, leptin decreases to levels below those expected for non-smokers of similar weight [32] and resting energy expenditure (REE) returns to normal [41].

In patients with RA, smoking has been shown to elevate REE [12]; however, no data are available on other potential contributors to smoking-related weight loss or smoking cessation-related weight gain for this population. Although we did not assess energy intake and expenditure or related regulators (such as leptin), it is likely that the mechanisms behind the reduced body weight of current smokers and the increased body weight of ex-smokers with RA are similar to those described for the general population.

Interestingly, the lower BMI of current smokers in the present study seems to be due to decreased BF rather than FFM. A possible mechanism by which smoking may affect fat metabolism is through a reduction in neuropeptide Y. This molecule not only stimulates food intake, but also promotes white fat lipid storage and decreases brown fat thermogenesis [35], so its inhibition through smoking would be expected to have the opposite effects. Additionally, smoking results in decreased adipose tissue lipoprotein lipase activity [42], which diverts fat storage away from adipose tissue and toward utilization by muscle [43], possibly leading to the decreased BF of smokers [42,44]. In the present study, the inverse association between smoking and BF appeared to be dose-dependent: increasing pack-years associated with reducing BF levels. Smoking cessation is thought to result in a reversal of the mechanisms described above, leading to increases in BF [42] and, most importantly, abdominal fat [45]. Indeed, among these RA patients, ex-smokers seemed to be the most 'unhealthy' group in terms of body weight and composition as they exhibited the highest BMI, BF, and waist circumference values.

In predominantly healthy people who are from the general population and who do not have wasting muscle disease, smoking of any intensity has been implicated in muscle wasting [10] by impairing the process of muscle protein synthesis [46]. In contrast, in the present study, only very heavy smoking appeared to associate with a reduction in FFM. It is possible that the effect of smoking on muscle is of less significance than the muscle loss associated with RA itself, as part of rheumatoid cachexia. This hypothesis is supported by the finding that increased duration of smoking (that is, pack-years) associated with lower FFM in both current and ex-smokers, which suggests the existence of a threshold below which smoking does not induce further muscle loss in RA patients. A longitudinal study of the impact of smoking intensity (and cessation) on the body composition of patients with RA may throw more light on the mechanistic basis of these observations.

Overall, this study suggests that, in RA, smoking associates with reduced body mass and fatness without inducing further muscle loss, except in very heavy smokers; in contrast, smoking cessation associates with increased body mass and fatness. This should not be interpreted as favouring what is a very unhealthy habit. Smoking cessation, even if it occurs in mid-life, reduces most of the later risk of death from tobacco [47]. However, smoking cessation is known to result in body weight increase, and this may affect some people's decision to stop smoking [11,44,45]. Therefore, any smoking cessation regime should be underpinned by more generalised lifestyle counselling, including advice on exercise and weight management. This is emphasized by the fact that, based on recently described RA-specific BMI [5], BF [16], and waist circumference thresholds [48], ex-smokers have the highest prevalence of obesity – both total and abdominal. FFM did not differ between groups and the prevalence of low FFM was comparable to that expected in age- and gender-matched healthy individuals [18].

## Conclusion

Within the limitations of this study, it is concluded that RA smokers have a lower BMI and BF than RA non-smokers, while heavy smokers also have a reduced FFM. A history of smoking cessation appears to associate with increases in BMI, BF, and waist circumference. Nevertheless, given the numerous adverse effects of smoking on health, smokers with RA should be actively advised against it, but smoking cessation programs should include wider lifestyle counselling for weight control, also focusing on increased physical activity and a healthy diet.

## Abbreviations

ANCOVA = analysis of covariance; ANOVA = analysis of variance; BF = body fat; BMI = body mass index; CI = confidence interval; CRP = C-reactive protein; DAS28 = Disease Activity Score-28; ESR = erythrocyte sedimentation rate; FFM = fat-free mass; HAQ = Health Assessment Questionnaire; RA = rheumatoid arthritis; REE = resting energy expenditure.

## Competing interests

The authors declare that they have no competing interests.

## Authors' contributions

AS-K participated in patient recruitment, data collection and analysis, and the drafting of the manuscript. GSM participated in patient recruitment and in data collection and analysis. VFP and KMJD participated in patient recruitment, rheumatological clinical assessments, and application of diagnostic/classification criteria. AMN provided expert statistical advice and supervision and participated in the review of the manuscript. AZJ participated in the inception and development of protocol and in the review of the manuscript and served as PhD program supervisor. MK provided advice on protocol development and body composition assessments and participated in the review of the manuscript. YK participated in the inception and development of protocol and served as PhD program supervisor. GDK participated in the inception and development of protocol, patient recruitment, clinical assessments, and analytical approach, provided supervision in the drafting of the manuscript, and served as PhD program supervisor and study guarantor.
